# Anger Weakens Behavioral Inhibition Selectively in Contact Athletes

**DOI:** 10.3389/fnhum.2018.00463

**Published:** 2018-11-20

**Authors:** Xue Xia, Guanghui Zhang, Xiaochun Wang

**Affiliations:** ^1^School of Kinesiology, Shanghai University of Sport, Shanghai, China; ^2^Department of Mathematical Information Technology, University of Jyväskylä, Jyväskylä, Finland

**Keywords:** athlete, anger, behavioral inhibition, evoked delta, evoked theta

## Abstract

Studies have increasingly found that the aggression level of contact athletes is higher than that of non-athletes. Given that higher aggression levels are associated with worse behavioral inhibition and that athletes show better behavioral inhibition than non-athletes, it is unclear why contact athletes would exhibit higher aggression levels. Emotion, especially anger, is an important factor in the generation of aggressive behavior, and anger has been shown to affect behavioral inhibition. Thus, the present study examined the influence of anger on behavioral inhibition in contact athletes. An implicit emotional Go/No-go task was used that contained 50 anger-associated words and 50 neutral words as stimuli. Participants were asked to execute a key press depending on the explicit color of word and to ignore any (implicit) emotional information associated with the word. The results showed a significant interaction in performance accuracy on the No-go task between emotion (i.e., anger-associated words versus neutral words) and group (athlete versus non-athlete). The performance accuracy of the contact athletes on anger-associated stimuli was significantly lower than that for neutral stimuli. Evoked delta and theta oscillations were analyzed at the time windows 200–600 and 200–400 ms respectively in both groups. A time-frequency analysis indicated a significant interaction between group, emotion and task for both evoked delta and theta oscillations. *Post hoc* analyses showed that stronger evoked delta and theta oscillations were evoked during the presentation of anger-associated stimuli compared with neutral stimuli on the No-go task in athletes. By contrast, no other significant effect was found in the control group or between the control and athlete groups. These results indicate that time-frequency analysis can effectively distinguish conventional ERP components and that implicit anger significantly weakens behavioral inhibition in contact athletes but not in non-athletes.

## Introduction

Physical or verbal conflicts are appearing in various sports with increasing frequency. When an athlete deliberately attacks others, it constitutes aggressive behavior, an intentional act meant to cause harm (Bushman and Anderson, 2001). Studies comparing the levels of aggressive behavior between athletes and non-athletes found that athletes shown higher aggression level especially high-contact athletes (Young, 1990; O’Brien et al., 2012; Sonderlund et al., 2014; Rui and Cruz, 2017). Although these studies have revealed higher aggression levels among athletes, the reasons for this increased aggression remain unclear. Researchers found that individuals with high aggression or impulsivity levels tend to have poor behavioral inhibition (Vigilcolet and Codorniuraga, 2004; Chen et al., 2005; Krämer et al., 2011; Pawliczek et al., 2013; Dambacher et al., 2015; Messerotti et al., 2015; Micai et al., 2015; Verona and Bresin, 2015). These results have suggested that the poorer the individual’s behavioral inhibition ability, the easier it is to initiate aggressive behavior. However, it has also been consistently demonstrated that athletes show better behavioral inhibition than non-athletes (Kida et al., 2005; Chan et al., 2011; Wang et al., 2013; Jacobson and Matthaeus, 2014). Behavioral inhibition, meaning the inhibition of a pre-potent response, is an important executive function in human behavior (Aron, 2007; Bari and Robbins, 2013). This leads to the question of why athletes would be more aggressive if they have better behavioral inhibition.

A model integrating multiple theories of aggression, the General Aggression Model (Anderson and Bushman, 2002), highlights the importance mood and emotion play in aggressive behavior. In addition, a recent study found that only anger, not general negative emotion, predicted self-reported aggression (Wyckoff, 2016). Thus, anger appears to be a critical factor in aggressive behavior (Denny and Siemer, 2012). Through the dimensional theory of emotion, anger is always considered a negative emotion (Reisenzein, 1992; Barrett, 2006).

A great deal of research using an emotional Go/No-go task has revealed that negative emotion can weaken an individual’s behavioral inhibition (Shafritz et al., 2006; Goldstein et al., 2007; Verona et al., 2012; Yu et al., 2014). In the Go/No-go task, participants are asked to respond to one stimulus (Go task) and to withhold the response to another stimulus. This task has been widely used in combination with the recording of event-related potentials (ERPs). Two conventional ERP components, N2 and P3, have been associated with behavioral inhibition. The No-go N2 component located in the right hemisphere lateral orbitofrontal and cingulate cortices at 200–300 ms after the No-go stimulus has been presented is thought to reflect conflict monitoring (Bokura et al., 2001; Donkers and Boxtel, 2004). The No-go P3 component located in left hemisphere lateral orbitofrontal cortex 300–600 ms after the No-go stimulus presentation represents the inhibition of the motor system (Bokura et al., 2001; Smith et al., 2008, 2013). An emotional task proven to be effective for exploration of behavioral inhibition (Schulz et al., 2007) contains two versions: one is explicit and the other is implicit. The emotional information of the stimuli is considered the Go and No-go targets in the explicit task. By contrast, gender, facial expression, or word color is the target in the implicit task, with the emotional information processing occurring implicitly. One study compared explicit and implicit emotional tasks with facial expression as the stimulus (Yu et al., 2014). The results revealed a significantly larger amplitude of the No-go P3 component and a higher current source density of right inferior frontal junction for sad expressions compared with that for neutral expressions in explicit tasks rather than implicit tasks, which represent a more cognitive resource. This result indicates that sadness can influence behavioral inhibition through modulating attention resources in the active inhibition stage, but it also demonstrated that there was a disassociation between explicit and implicit emotional tasks. Using an implicit emotional Go/No-go task, researchers found that 4–6-year-old children displayed larger amplitude of the No-go N2 component for angry facial expressions than for neutral expression, indicating that when processing anger information, children need more attentional monitoring and cognitive resources (Todd et al., 2008). Together, these studies underscore the influence of emotion on behavioral inhibition.

Regarding time-domain measures of ERPs, researchers have suggested that the conventional ERP components are unable to reflect separable but overlapping processes (Harper et al., 2014). However, time-frequency analysis approaches can effectively separate the time-frequency components that represent the underlying processes (Karakaş et al., 2000a; Başar-Eroglu and Demiralp, 2001; Harmony et al., 2009; Bernat et al., 2012). Researchers found that both delta and theta oscillation band frequencies were appeared in both of the N2 and P3 component time windows in a Go/No-go task (Karakaş et al., 2000b; Kirmizi-Alsan et al., 2006; Harper et al., 2014; Messerotti et al., 2017). Enhanced delta activity is thought to reflect motor inhibition, similar to the P3 component, and the theta activity in the No-go task is thought to reflect initial conflict monitoring during response execution and suppression, similar to the N2 component (Huster et al., 2013; Harper et al., 2014).

Hence, the present study aimed to explore the influence of anger on behavioral inhibition in athletes and non-athletes with an emotional Go/No-go task to help determine why athletes show higher aggression. Because the stimulus materials have certain emotionality, if an explicit task is used, the participants will react according to the emotionality, which will lead them to understand the purpose of the experiment and the experimental intent of the main test. Therefore, an implicit task was used in this study. We also aimed to resolve the traditional time-domain N2 and P3 components by using time-frequency analyses. We hypothesized that athletes would show poor behavioral inhibition in response to stimuli associated with anger, which would suggest that anger weakens behavioral inhibition in athletes.

## Materials and Methods

### Participants

In total, 30 undergraduate students participated in the experiment. Fifteen athletes comprised the athlete group, and 15 non-athletes comprised the control group. All 15 athlete participants were national second-level athletes, with sports experience of 7 to 10 years in basketball or football, eight were men, and the mean age of the group was 20.1 years (*SD*: 1.5 years). These two sports were chosen because they are contact sports with players having been previously found to be susceptible to emotion and aggression (Uphill et al., 2014; Rui and Cruz, 2017; Cho et al., 2018; Patricios et al., 2018). The control group was age- and gender-matched to the athletes. All participants were right-handed with normal or corrected-to-normal vision and were recruited from Shanghai University of Sport. This study was conducted in accordance with recommendations of the World Medical Association’s Declaration of Helsinki. The study was approved by Shanghai University of Sport Ethics Committee (Shanghai, China), and written informed consent was obtained from all participants. After finishing the experiment, each participant was financially compensated with 50 RMB.

### Questionnaire

To assess the trait anger of athletes and non-athletes, participants completed the State-Trait Anger Expression Inventory 2 (Spielberger, 1988) before the experiment. Trait anger was assessed by the trait anger scale. The score showed that there was no significant difference in trait anger between athletes (mean: 18.13; *SD*: 4.36) and non-athletes (mean: 17.20; *SD*: 4.72; *P* = 0.578). State anger was assessed using the state anger scale. The score on this scale showed that there was no significant difference in state anger between athletes (mean: 16.27; *SD*: 2.28) and non-athletes (mean: 17.27; *SD*: 4.57; *P* = 0.457).

### Stimuli and Procedure

The stimuli presented in the experiment included 110 words: 50 words associated with anger and 50 neutral words for the formal experiment; 10 additional words served as stimuli for participant practice. All words were chosen from the Chinese Affective Words System based on the valence value. Prior to beginning the formal experiment, 200 words were selected for a pilot study, and 49 college students not participating in the formal experiment were asked to rate how anger they felt about the words using a Likert scale with anchors of 1 (not angry at all) and 9 (extremely angry) and how arousal they felt about the words also from 1 (lowest arousal) to 9 (highest arousal). The 50 highest scoring words were classified as words associated with anger (valence mean, 6.13 ± 0.22; arousal mean, 3.02 ± 0.19), and the 50 lowest scoring words were classified as neutral (valence mean, 2.93 ± 0.15; arousal mean, 2.75 ± 0.31). The valence of the anger for words associated with anger was significantly greater than that for the neutral words, whereas these types of words showed equal arousal; thus, the two types of words differed only in the valence of the associated anger.

**FIGURE 1:**
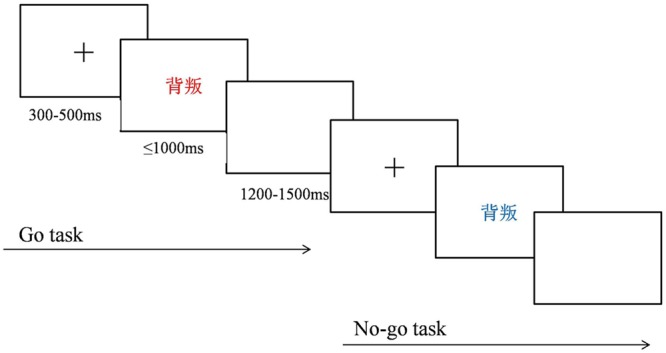
Emotional Go/No-go task procedure.

An implicit version of the emotional linguistic Go/No-go task was used and was programmed with E-Prime 2.0. Similar to that in previous studies, the emotional information was task-unrelated. The Go and No-go stimuli were words colored either blue or red. Participants were instructed to press a key on a keyboard with their right index finger as quickly and accurately as possible when a red-colored word appeared on a computer screen (Go trial) and to inhibit this response, if the word was colored blue (No-go trial). At the beginning of each trial, a central fixation cross appeared for 300–500 ms. A word was then presented for 1000 ms (No-go trial) or disappeared with the key press within 1000 ms (Go trial). A blank screen was then presented for 1200–1500 ms at the end of the trial. The task consisted of four blocks of 100 stimuli per block. Each block contained 75 Go trials (red-colored words), creating a pre-potent response tendency, and 25 No-go trials (blue-colored words), for examination of response inhibition, with 50 words associated with anger and 50 neutral words randomly appearing with the same probability. Color plays an important role in cognition, especially affecting the cognitive processes of emotional perception, time perception, and aggressive behavior (Codispoti et al., 2011; Guéguen et al., 2012; Shibasaki and Masataka, 2014). Because these cognitive processes may influence the results of the present study, the colors for the Go or No-go stimulus were counterbalanced between participants. The task procedure is illustrated in Figure 1.

### Behavioral Data Analysis

Behavioral data that were more than three standard deviations from the mean were discarded. The remaining behavioral data were imported into SPSS, version 20.0. The accuracy on Go trials was excluded from the analysis because these data are subject to a ceiling effect (Mennella et al., 2017). A repeated measures analysis of variance (ANOVA) was used to analyze reaction times for correct Go trials and accuracy for correct No-go trials, with group (athlete and control) as the between-subject factor and emotion (anger and neutral) as the within-subject factor. Values of *P* (two-tailed) less than 0.05 were considered statistically significant. Greenhouse–Geisser corrections were used for within-subject factors and interactions when applicable.

### Electrophysiologic Data Acquisition and Analysis

Electroencephalography (EEG) activity was recorded using the BrainVision Recorder 2.0 system (Brain Products GmbH, Germany) with an electrode cap containing 64 sintered Ag-AgCl electrodes placed according to the International 10–20 system. The EEG data were referenced online against the FCz electrode and grounded at the AFz electrode. A vertical electrooculogram was obtained below the left eye, and the horizontal electrooculogram was obtained at the outer canthus of the right eye. The data sampling rate was 500 Hz, with 0.01–100 Hz bandpass filtering by a BrainAmp amplifier. Electrode impedance was maintained below 5k ohm during the experiment.

For time-domain analysis, first, the reference electrode was converted offline to both posterior ear papillae (TP9 and TP10 electrodes) (Debener et al., 2012), and the FCz electrode was restored using the Analyzer 2.0 system (Brain Products). The data were then preprocessed in MATLAB and with the EEGLAB toolbox of Delorme and Makeig (2004). This analysis included the use of a 50 Hz notch filter, to remove line noise, as well as filtering (0.2 Hz high-pass filter, 30 Hz low-pass filter), segmentation of the filtered continuous EEG into single trials (each trial was extracted offline from 200 ms prestimulus onset to 1000 ms post-stimulus onset), baseline correction using the 200 ms preceding cue onset, artifact rejection of amplitudes exceeding ± 100 μV, removal of trials contaminated by eye blinks with independent component analysis, and averaging. Previous studies have found that the N2 component is a negative shift between 200 and 300 ms and the P3 component is a positive shift between 300 and 600 ms in the Go/No-go task. In addition, the difference wave (Nogo- minus Go- ERPs) is better to present the Go/No-go effect (Liu et al., 2014). Thus, combined with the waveform in present study, repeated measurements of emotion (anger and neutral) as within-subject factors and group (athlete and control) as between-subject factors was conducted on the difference wave of N2 component at the F2, F4, FC2, and FC4 electrodes between 200 and 300 ms, and the difference wave of P3 component at the FCz, Cz, and CPz electrodes between 300 and 500 ms by calculating the average amplitude.

In present study, time-frequency analysis was used to resolve traditional time-domain ERP components to acquire the oscillation responses at different time scales or frequencies which are known to represent overlapping delta and theta activities of several neural networks during behavioral inhibition (Ergen et al., 2014). Two methods of time-frequency analysis are generally used. In the first, the data are analyzed after averaging to acquire time-locked and phase-locked potentials, called evoked event-related oscillations (EROs) which synchronized with the event. In the second method, the data from each single trial are analyzed, and then the average of the results of each single trial is determined to acquire phase-locked and non-phase-locked potential. This potential is the total activity contained in both the evoked and induced EROs (Herrmann et al., 2004; Herrmann et al., 2014). As ERP is mainly generated by the phase modulation mechanism and the stimulus transforms the spontaneously EEG oscillation phase resetting into a synchronous oscillation (Sayers et al., 1974). Thus, the synchronous oscillation is much better to resolving the ERP component. In addition, the ERPs are complied with the principle of superposition, the analysis of the compound ERP waveform should be composed of the oscillation response as the unit of analysis (Karakaş et al., 2000b). Therefore, evoked EROs were analyzed in the presented study to disentangle the multiple processes underlying time-domain ERP data. A complex Morlet continuous wavelet transform (CMCWT) based on the complex wavelet transform (Kronland-Martinet et al., 1987; Tallon-Baudry et al., 1996; Demiralp et al., 2001) was used for time-frequency (TF) analysis of the average ERP data in the MATLAB toolbox. CMCWT was described as *CMCWT (t, f) =* |(MCt,f)^∗^x(t)|^2^. The time-frequency energy CMCWT (t, f) was used to calculate the convolution of the mother wavelet $\Phi (t,f_{c})=\frac{1}{\sqrt{\pi \sigma^{2}}}e^{i2\pi tf_{c}}e^{\frac{-t^{2}}{2\sigma^{2}}}\;$ (f_c_, center frequency; σ, bandwidth). A wavelet family was characterized by the constant ratio $\;K=\frac{f_{c}}{\sigma_{f}}=2\pi \sigma f_{c}$ with K being greater than 5 (Zhang et al., 2017). A baseline correction using the 200 ms preceding cue onset again was then conducted.

Combined the previous study (Pandey et al., 2015) and present data, the FCz, Cz, and CPz electrodes were selected for the analysis of the evoked delta oscillation (0.5–3.5 Hz) between 200 and 600 ms, and the Fz, FCz, and Cz electrodes were selected for the analysis of evoked theta oscillation (4–7 Hz) between 200 and 400 ms. Multivariate repeated measures ANOVAs were used to analyze evoked delta and theta oscillations, with emotion (anger and neutral), task (Go and No-go) as within-subject factors and group (athlete and control) as between-subject factors.

## Results

### Behavior

There was no significant main effect of group or emotion and no significant interaction for reaction times between group and emotion in the correct Go trials. The descriptive statistics of reaction times is given in Table 1.

**Table 1:** Reaction times in the Go task.

Emotion	Athlete		Control	
	Anger	Neutral	Anger	Neutral
Mean (ms)	349.093	346.177	348.513	349.387
*SD*	35.080	38.542	46.371	43.920

**FIGURE 2:**
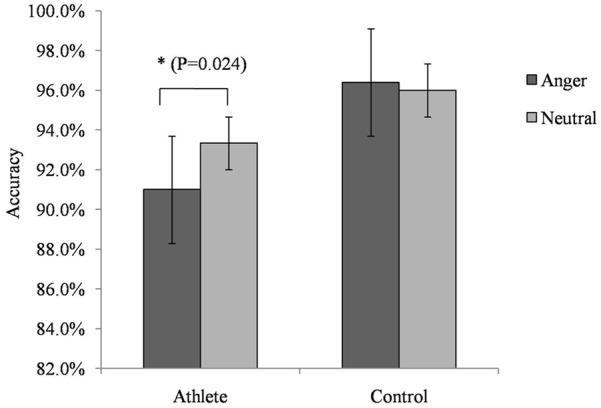
Accuracy on the No-go task during presentation of anger-associated and neutral stimuli among athletes and non-athlete controls. ^∗^p < 0.05.

For accuracy in the No-go trials, we found a significant interaction between group and emotion [*F*_(1,28)_ = 4.735; *P* = 0.038; $\eta_{p}^{2}$ = 0.145]. A simple effects analysis showed that the accuracy of athletes on trials with anger-associated stimuli (mean: 0.915; *SD*: 0.018) was significantly lower than that on trials with neutral stimuli (mean: 0.933; *SD*: 0.018; *P* = 0.024) (Figure 2). No other significant behavioral effect was detected.

### Time-Domain Analysis

**FIGURE 3:**
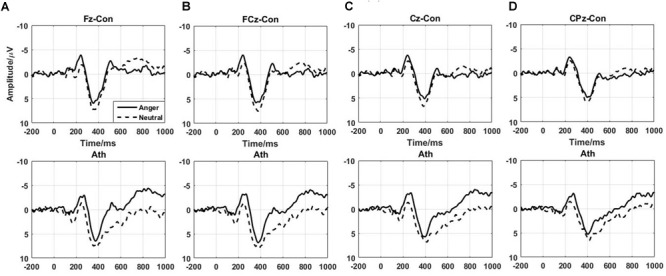
Time-domain ERPs of the No-go minus Go differences at the Fz, FCz, Cz, and CPz electrodes in athlete and control groups. Ath, athlete; Con, control. **(A)** Fz electrode; **(B)** FCz electrode; **(C)** Cz electrode; **(D)** CPz electrode.

**FIGURE 4:**
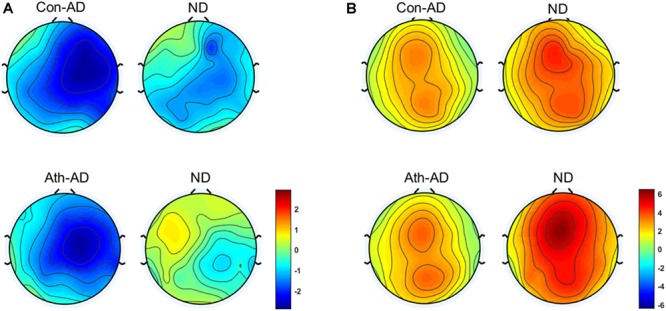
Topographical distribution of N2 and P3 component. **(A)** N2 at 200–300 ms; **(B)** P3 at 300–500 ms. Ath, athlete; Con, control; AD, Difference waveform of anger-associated stimuli; ND, Difference waveform of neutral stimuli.

The difference waves of time-domain N2 and P3 components are shown in Figure 3 with the topographical distribution are shown in Figure 4. Here, we only conducted double-factor repeated measurements of emotion (anger and neutral) and group (athlete and control) on the difference waves representing the Go/No-go effect, which contributed to a clearer presentation of the role of emotion in different subjects (Bo et al., 2009; Liu et al., 2014). We found that the time-domain N2 was a negative deflecting waveform with the maximum peak between 200 and 300 ms, whereas the P3 component was a positive deflecting waveform with a maximum peak between 300 and 500 ms. Considering the strong lateralization in N2 component and more symmetry in P3 component in present study, the F2, F4, FC2, and FC4 electrodes were chosen for the analysis of N2 component, and the FCz, Cz, and CPz electrodes were chosen for the analysis of P3 component. Repeated measures ANOVAs of N2 component indicated that significant main effect was found in the emotion condition [*F*_(1,28)_ = 7.435; *P* = 0.011; $\eta_{p}^{2}$ = 0.210] with larger amplitude of anger condition than neural condition. Whereas no significant main effect was found in the group [*F*_(1,28)_ = 0.959; *P* = 0.336; $\eta_{p}^{2}$ = 0.033] or interaction between group and emotion [*F*_(1,28)_ = 0.654; *P* = 0.426; $\eta_{p}^{2}$ = 0.023]. Repeated measures ANOVAs of P3 component also indicated that significant main effect was found in the emotion condition [*F*_(1,28)_ = 9.886; *P* = 0.004; $\eta_{p}^{2}$ = 0.261] with larger amplitude of neural condition than anger condition. Whereas no significant main effect was found in the group [*F*_(1,28)_ = 1.271; *P* = 0.269; $\eta_{p}^{2}$ = 0.043] or interaction between group and emotion [*F*_(1,28)_ = 0.503; *P* = 0.484; $\eta_{p}^{2}$ = 0.018].

### Time-Frequency Analysis

**FIGURE 5:**
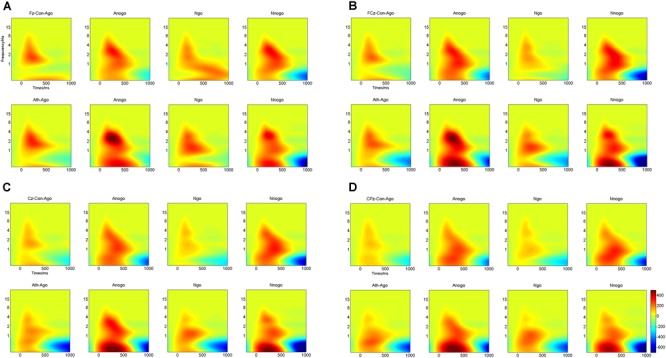
Time-frequency representations of evoked delta and theta oscillations at four electrodes for athletes and controls. **(A)** Fz electrode; **(B)** FCz electrode; **(C)** Cz electrode; **(D)** CPz electrode. Ath, athlete; Con, control; Ago, go task of the anger-associated words; Anogo, no-go task of the anger-associated words; Ngo, go task of the neutral words; Nnogo, no-go task of the neutral words.

Multivariate repeated-measures ANOVAs were computed on evoked delta oscillation and evoked theta oscillation using emotion (anger and neutral), task (Go and No-go) as within-subject factors and group (athlete and control) as between-subject factors. The time-frequency representations of evoked delta and theta frequency oscillation at the Fz, FCz, Cz, and CPz electrodes are illustrated in Figure 5.

#### Evoked Delta Oscillation

**FIGURE 6:**
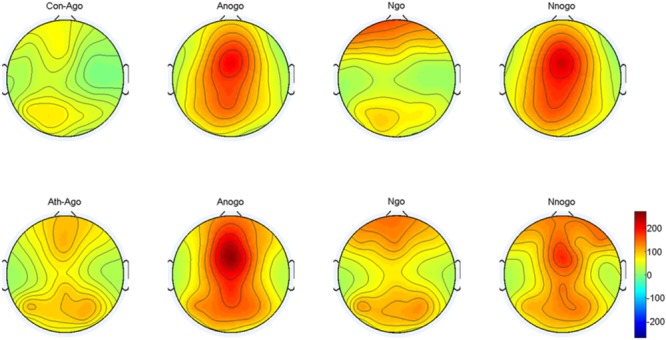
Topographical distribution of the evoked delta oscillations at 200–600 ms. Ath, athlete; Con, control; Ago, go task of the anger-associated words; Anogo, no-go task of the anger-associated words; Ngo, go task of the neutral words; Nnogo, no-go task of the neutral words.

The topographical distribution of the evoked delta oscillation (0.5–3.5 Hz) is shown in Figure 6. Overt evoked delta oscillations were present at 200–600 ms in both emotional conditions (anger-associated and neutral stimuli) and groups (athletes and controls), especially in the No-go task. Based on the topographical distribution, evoked delta power was analyzed between 200 and 600 ms at the FCz, Cz, and CPz electrodes.

The result showed a significant main effect of task [*F*_(1,28)_ = 14.315; *P* = 0.001; $\eta_{p}^{2}$ = 0.338]. The evoked delta power was significantly higher in No-go task than Go task which further suggested that evoked delta was associated with response inhibition. In addition, there was a significant interaction between group and emotion [*F*_(1,28)_ = 4.488; *P* = 0.043; $\eta_{p}^{2}$ = 0.138]. The simple effects analysis revealed a significant effect for emotion (*P* = 0.033) only in the athlete group, with the evoked delta power stronger for anger-associated stimuli than for neutral stimuli. By contrast, there was no significant difference in evoked delta power for anger-associated and neutral stimuli in the control group (*P* = 0.460). Moreover, we found a significant interaction of these three factors [*F*_(1,28)_ = 4.599; *P* = 0.041; $\eta_{p}^{2}$ = 0.141] which indicated the further interaction of emotion with group and task. *Post hoc* analyses showed that in the athlete group, the evoked delta power was significantly higher for anger-associated than neutral stimuli in the No-go task (*P* = 0.016), not in the Go task (*P* = 0.846). Whereas there was no significant difference in evoked delta power for anger-associated and neutral stimuli in control group whether in the No-go task (*P* = 0.508) or Go task (*P* = 0.689). And no other interaction effects or main effects were found in the power of evoked delta. The evoked delta power for the two groups in the 200–600 ms time window is given in Table 2.

**Table 2:** Evoked delta power of the two groups in the 200–600 ms time window.

Emotion	Athlete				Control			
	Anger		Neutral		Anger		Neutral	
	Go	No-go	Go	No-go	Go	No-go	Go	No-go
Mean (μV^2^)	79.529	213.074	81.451	150.139	36.468	179.431	40.427	195.841
*SE*	25.719	38.435	27.525	38.894	15.024	45.397	13.536	50.777

#### Evoked Theta Oscillation

**FIGURE 7:**
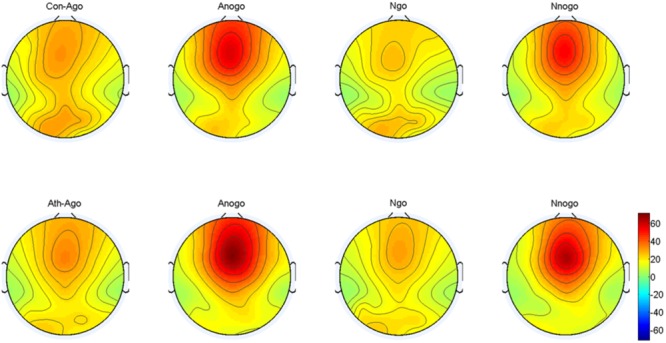
Topographical distribution of the evoked theta oscillations at 200–400 ms. Ath, athlete; Con, control; Ago, go task of the anger-associated words; Anogo, no-go task of the anger-associated words; Ngo, go task of the neutral words; Nnogo, no-go task of the neutral words.

The topographical distribution of the evoked theta oscillation (4–7 Hz) is shown in Figure 7. Overt evoked theta oscillations were present at 200–400 ms in both emotional conditions (anger-associated and neutral stimuli) and groups (athletes and controls), especially in the No-go task. Based on the topographical distribution, evoked theta power was analyzed between 200 and 400 ms at the Fz, FCz, and Cz electrodes.

The result showed a significant main effect of task [*F*_(1,28)_ = 33.748; *P* = 0.000; $\eta_{p}^{2}$ = 0.547] and emotion [*F*_(1,28)_ = 7.025; *P* = 0.013; $\eta_{p}^{2}$ = 0.201]. The evoked theta power was significantly higher in No-go task than Go task which also suggested that evoked theta was associated with response inhibition. And significant higher evoked theta power for anger-associated stimuli than neutral stimuli. In addition, there was a significant interaction between group and emotion [*F*_(1,28)_ = 6.410; *P* = 0.017; $\eta_{p}^{2}$ = 0.186]. The simple effects analysis revealed a significant effect for emotion (*P* = 0.001) only in the athlete group, with the evoked theta power stronger for anger-associated stimuli than for neutral stimuli. By contrast, there was no significant difference in evoked theta power for anger-associated and neutral stimuli in the control group (*P* = 0.934). Moreover, a significant interaction between emotion, group and task [*F*_(1,28)_ = 5.572; *P* = 0.025; $\eta_{p}^{2}$ = 0.166] was shown. *Post hoc* analyses showed that in the athlete group, the evoked theta power was significantly higher for anger-associated than neutral stimuli in both No-go task (*P* = 0.004) and Go task (*P* = 0.017). Whereas there was no significant difference of evoked theta power between different emotional stimuli in control group whether in the No-go task (*P* = 0.678) or Go task (*P* = 0.062). And no other interaction effects or main effects were found in the power of evoked theta. The evoked theta power for the two groups in the 200–400 ms time window is given in Table 3.

**Table 3:** Evoked theta power of the two groups in the 200–400 ms time window.

Emotion	Athlete				Control			
	Anger		Neutral		Anger		Neutral	
	Go	No-go	Go	No-go	Go	No-go	Go	No-go
Mean (μV^2^)	115.652	318.201	93.325	211.535	76.573	205.670	59.429	219.860
*SE*	31.548	52.708	31.345	44.271	18.018	29.102	31.345	39.530

Thus, the present study has found that at the behavioral level, the accuracy on anger words of Go task was significantly lower than neutral words in athletes while the non-athletes did not show this difference. At the neural level, the evoked delta and theta power significantly increases in anger words when compared to neutral words of the No-go task which also only in the athletes.

## Discussion

The present study used an implicit emotional Go/No-go task to investigate whether anger differentially influences athletes compared with non-athletes to explain the higher aggression level associated with athletes. At first, we excluded the effect of trait anger on behavioral inhibition (Liu et al., 2014; Lievaart et al., 2016). Then participants were asked to respond to the color of a presented word and to ignore any emotional information (implicitly processed). At the behavioral level, we anticipated finding significant differences in reaction times and accuracy between the athlete group and non-athlete control group for anger-associated stimuli versus neutral stimuli. At the neural level, we postulated that there would be significant differences in evoked delta and theta oscillations between the two groups for the two emotional conditions.

At the behavioral level, the accuracy of participants to perform No-go trials reflects an individual’s behavioral inhibition ability. We found that the accuracy on the No-go trials for anger-associated stimuli was lower than that for neutral stimuli only in the athlete group. Thus, we believe that when processing anger information, behavioral inhibition in athletes is decreased, making it more difficult for athletes to ignore anger-associated stimuli. However, reaction times on Go trials were similar for both groups and for both types of stimuli. This finding may be associated with a ceiling effect (Berchicci et al., 2015). The response target in the present study was the color of the word, meaning that participants merely needed to see the word color to execute or withhold a key press. This task may have been sufficiently easy for the participants to perform that the reaction times during the Go trials could not distinguish the two groups or the two emotional conditions.

At the neural level, we detected distinct time-domain N2 and P3 components as in previous studies (Wang et al., 2011; Ergen et al., 2014; Messerotti et al., 2015). The time window of 200–300 ms is in the range of the N2 component, which is thought to reflect signal detection and conflict monitoring in the early stage of behavioral inhibition. The time window of 300–500 ms is within the range of the P3 component, which is thought to represent motor inhibition in the late stage of behavioral inhibition. In present study, we found significant main effect of emotion conditions of the difference waves of the N2 and P3 component which indicated the effectiveness of emotional manipulation. However, as mentioned in previous studies, the ERP waveform overlaps in time and topographical distribution; thus, analyzing only one ERP component in a certain time window does not adequately reflect the entire process of information processing. This may be why that although we detected the main effect of emotion conditions, no significant differences between group and emotion were found in the present study. Thus, we resolved the traditional time-domain N2 and P3 components by using time-frequency analyses.

Using time-frequency analyses, we observed distinct evoked delta oscillations (0.5–3.5 Hz) and evoked theta oscillations (4–7 Hz) during the processing phase. Evoked delta activity has been associated with motor inhibition (similar to the No-go P3), whereas evoked theta activity has been associated with conflict detection between reaction execution and suppression (similar to the No-go N2) (Harper et al., 2014; Messerotti et al., 2015). Our results were consistent with these previous findings, confirming that conventional time-domain N2 and P3 components contained at least two processes that can be separated by time-frequency analysis with the evoked delta and theta activities contribute to these two components (Yordanova et al., 2000; Kirmizi-Alsan et al., 2006; Harper et al., 2014). We then conducted multivariate repeated measures ANOVA on the power of the evoked delta and theta oscillations at 200–600 ms and at 200–400 ms respectively. The results showed that for the athlete group, the power in the evoked delta and theta oscillations during the presentation of anger-associated stimuli were stronger than those for neutral stimuli in the No-go task, suggesting that when athletes executed behavioral inhibition during a stimulus associated with anger, greater evoked delta and theta activities were evoked. Greater evoked delta activity means that the athletes needed to put more effort into the process of suppressing the key press, and great evoked theta activity indicated greater conflict awareness during presentation of the anger-associated stimulus. We believe that it was precisely because of the decreased behavioral inhibition during the anger-associated stimulus presentation that more awareness and effort were needed to suppress the anger. Increased evoked delta and theta activities have also been associated with the motivational system (Knyazev, 2007, 2012) and emotion processing (Walden et al., 2015). The response tendency for an emotional stimulus may be reflected by the degree of difficulty in suppressing a pre-potent response. Therefore, the greater activation of the behavioral approach system and the lower activation of the behavioral inhibition system, the more difficult it is to withhold a response (Briggs and Martin, 2009; Messerotti et al., 2017). This is consistent with a characteristic of anger emotion, which is a negatively valenced emotion that typically evokes behavioral tendencies of approach (Harmon-Jones and Allen, 1998; Harmon-Jones, 2003; Carver and Harmon-Jones, 2009; Harmon-Jones et al., 2010). Athletes have shown greater difficulty in suppressing anger, indicating a lower activation of the behavioral inhibition system and a higher activation of the behavioral approach system, demonstrating decreased behavioral inhibition ability in athletes under anger-provoking conditions.

Notably, we detected no significant differences for anger-associated or neutral stimuli in the control group for both task at either the behavioral or neural levels. This may be due to the implicitness of this emotional Go/No-go task. A study comparing implicit and explicit emotional tasks found that the influence of emotion on inhibition was detected only in explicit but not implicit emotional tasks (Yu et al., 2014). This finding indicates that only when emotional intensity reaches a certain level will the individual’s behavioral inhibition ability be affected. In the present study, the Go or No-go task stimulus was the color of the word, with the emotional information processed incidentally. However, the implicit anger component significantly weakened the behavioral inhibition ability among athletes without significantly influencing non-athletes. This suggests that athletes are more sensitive than non-athletes to anger; even an anger-associated stimulus outside of conscious attention was sufficient to have a significant effect among athletes but not among non-athletes.

Unlike previous studies, we found that behavioral inhibition among athletes was not better than that among non-athletes (Kida et al., 2005; Chan et al., 2011; Wang et al., 2013; Jacobson and Matthaeus, 2014). However, unlike those previous studies, the Go/No-go task used in present study was an emotional task with implicitly processed anger-associated stimuli. An anger-associated or neutral stimulus appeared randomly in each block of trials, causing a mutual interference between the emotional effects (Pereira et al., 2006). In other words, the presentation of an anger-associated stimulus weakened the generally greater behavioral inhibition in athletes, and this negative effect may have continued into the next neutral stimulus presentation, resulting in an overall decrease in behavioral inhibition among the athletes. Thus, compared with non-athletes, athletes would show similar rather than better behavioral inhibition on this task.

## Conclusion

The present study used time-frequency analyses, which distinguished the time-domain N2 and P3 ERP components and better described the process of behavioral inhibition, to investigate the influence of anger on behavioral inhibition among contact athletes. We found that implicitly processed anger-associated stimuli significantly impaired behavioral inhibition among athletes, with athletes expending effort to inhibit anger. By contrast, the same anger-associated stimuli did not significantly affect non-athletes. These results suggest that poorer behavioral inhibition among athletes under anger-stimulating conditions makes it difficult for them to restrain their behavior, resulting in higher impulsivity and more aggressive behavior. Thus, strengthening behavioral inhibition among athletes under anger-stimulating conditions may help reduce their aggression and improve their performance on the field.

### Future Directions and Limitations

Although this study found that anger interfered with behavioral inhibition in athletes, we found no difference in any indicator between athletes and non-athletes. We believe that this is mainly because implicit anger is insufficient to weaken behavioral inhibition in non-athletes, whereas it is sufficient to interfere with the performance of athletes. In the present study, the anger-associated words were mixed with neutral stimuli, reducing the behavioral inhibition exhibited by athletes, which resulted in the same level of behavioral inhibition in both non-athletes and athletes. Therefore, future studies should explore how athletes and non-athletes react to the presentation of anger-associated stimuli separate from how they react to the presentation of neutral stimuli.

## Data Availability

The raw data supporting the conclusions of this manuscript will be made available by the authors, without undue reservation, to any qualified researcher.

## Author Contributions

XW and XX designed the experiments and wrote the paper. XX conducted the experiments. GZ and XX analyzed the data.

## Conflict of Interest Statement

The authors declare that the research was conducted in the absence of any commercial or financial relationships that could be construed as a potential conflict of interest.
